# Application of split pancreatic duct stent in laparoscopic pancreaticoduodenectomy

**DOI:** 10.1097/MD.0000000000034049

**Published:** 2023-08-04

**Authors:** Jianhua Tu, Changwen Huang, Wenyan Xu, Shuaichang Gong, Zhenjun Cao, Ping Wan, Junxiang Ying, Xuefeng Rao

**Affiliations:** a Nanchang University Medical College, Nanchang, China; b Department of Hepatobiliary Surgery, Jiangxi Provincial People's Hospital (The First Affiliated Hospital of Nanchang Medical College), Nanchang, China; c Qingyuan People’s Hospital, Qingyuan, China.

**Keywords:** pancreatic duct stent, pancreaticoduodenectom, postoperative pancreatic fistula

## Abstract

Laparoscopic pancreaticoduodenectomy (LPD) is a classic surgical method for diseases, such as tumors at the lower end of the common bile duct, pancreatic head, and benign and malignant tumors of the duodenum. Postoperative pancreatic fistula (POPF) is one of the most serious complications of LPD. To reduce the incidence of grade B or C POPF and other complications after LPD, we applied a split pancreatic duct stent combined with the characteristics of internal and external stent drainage. Between September 2020 and September 2022,12 patients underwent placement of the Split pancreatic duct stent during LPD. Data on basic characteristics of patients, surgical related indicators and postoperative POPF incidence were collected and analyzed. The results showed that the average operation time was 294.2 ± 36 minutes, average time for pancreaticojejunostomy was 35.9 ± 4.1 minutes, and average estimated blood loss was 204.2 ± 58.2 mL. Biochemical leakage occurred in 2 patients (16.7%), whereas no grade B or C POPF, 1 case (8.3%) had postoperative bleeding, and no death occurred within 30 days after the operation. Preliminary experience shows that the split pancreatic duct stent can effectively reduce the incidence of complications after LPD, especially grade B or C POPF.

## 1. Introduction

Pancreaticoduodenectomy is a classical surgical method for treating diseases, such as tumors at the lower end of the common bile duct, pancreatic head cancer, and benign and malignant tumors of the duodenum. With the introduction of laparoscopic technology in the 1980s, laparoscopic pancreaticoduodenectomy (LDP) has gradually been applied in clinical practice.^[1,2]^ Although LDP takes a longer time then open pancreaticoduodenectomy, it has the advantages of clear vision, less blood loss and shorter hospitalization time.^[3,4]^ Postoperative pancreatic fistula (POPF) is one of the most common and serious complications after LDP, with an incidence of approximately 5% to 40%, and a related mortality of approximately 1%.^[5–8]^ Risk factors for POPF include smoking, soft pancreas, high body mass index (BMI), blood transfusion, intraoperative blood loss and long operation time. To reduce the incidence of POPF, surgeons have adopted^[9]^ improved reconstruction methods (pancreatic stomach and pancreatic jejunum anastomoses),^[10]^ anastomosis techniques (catheter-mucosa and intrapped anastomoses),^[11]^ the use of stents (external or internal stents), and^[12,13]^ somatostatin and its analogs, and have achieved certain curative effects.^[14,15]^

Placement of stent in pancreaticojejunostomy (PJ) for internal or external drainage of pancreatic fluid can effectively prevent pancreatic fluid corrosion during anastomosis and reduce the incidence of POPF.^[16,17]^ Internal drainage can prevent digestive fluid loss and maintain gastrointestinal function stability, but there are problems such as early pancreatic juice reflux and poor observation of drainage.^[18,19]^ External drainage can drain more pancreatic juice to completely prevent residual pancreatic erosion, but there are complications such as late anastomotic collapse and skin ulceration around the drainage tube. In order to effectively combine the characteristics of internal and external drainage, we applied an external-to-internal drainage stent to reduce the incidence of postoperative B or C-level POPF and related severe complications.

## 2. Methods

### 2.1. Patients

We enrolled 12 patients who underwent LDP at the Department of Hepatobiliary Surgery, Jiangxi Provincial People’s Hospital, between September 2020 and September 2022 due to pancreatic head, lower common bile duct, or duodenal malignant tumors. patients’ general information, such as age, sex, BMI and American Society of Anesthesia scores, were collected. The following data were collected for each patient: operative time, duration of PJ, intraoperative blood loss, hospitalization time, postoperative first exhaust time, pathological diagnosis, mortality, POPF and other complications. This study was approved by the medical ethics committee of our hospital.

### 2.2. Surgical approach

**Figure 1.**
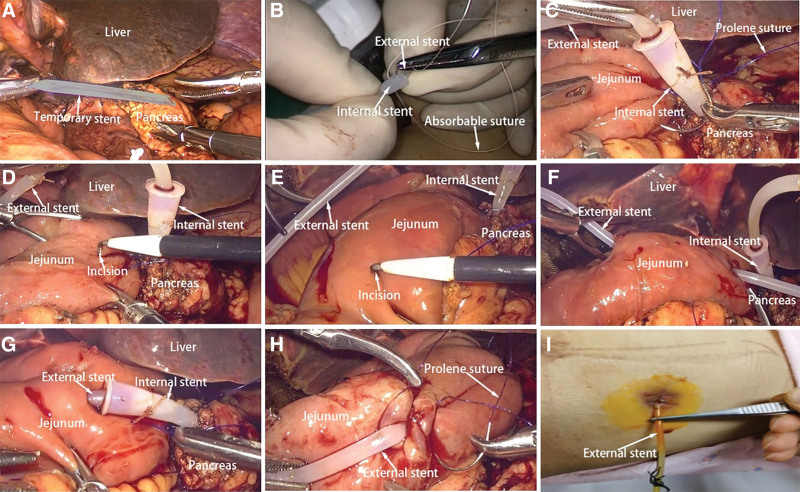
Practice chart of split pancreatic duct stent. (A) Removal of common pancreatic duct stent. (B) Fabrication of split pancreatic duct stent. (C) Anastomosis of posterior wall of PJ. (D) A coarse incision of the size of the internal stent was made at the proximal jejunum. (E) A small incision of the size of the external stent was made at the blind end of the jejunum. (F) The external stent was drawn through the jejunum from the small incision. (G) Internal stent introduced into jejunum. (H) Fix the external stent in the jejunum. (I) Removal of external stent. PJ = pancreaticojejunostomy.

**Figure 2.**
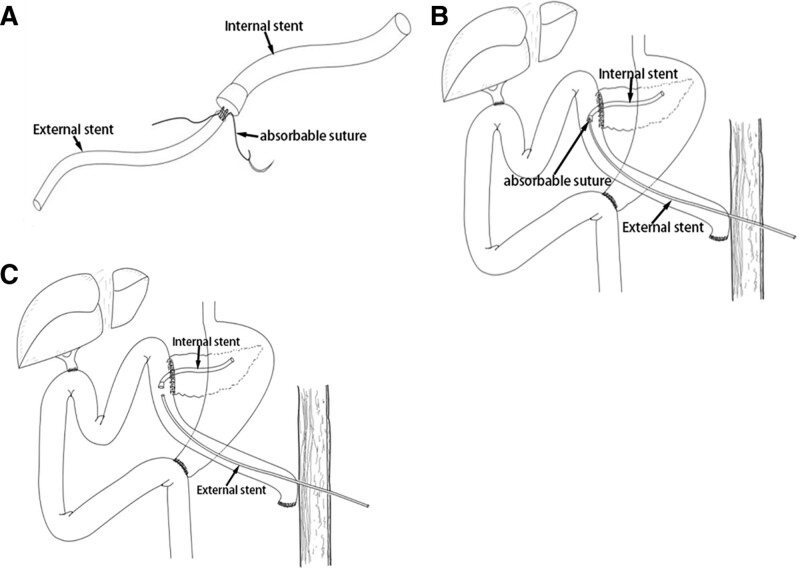
Simulation of split pancreatic duct stent. (A) Fabrication of the split pancreatic duct stent. (B) Placement of the split pancreatic duct stent. (C) Removal of the external stent part of the split pancreatic duct stent.

Relevant videos can be found in Supplementary Materials (Videos, Supplemental Digital Content, https://links.lww.com/MD/J374). The surgical operation layout adopted the conventional “five-hole method.” LPD was performed after exploration of the abdominal cavity and removal of peritoneal and other organ surface metastases. After the pancreas was excised, a common pancreatic duct stent was temporarily placed to find the pancreatic duct and estimate its size and depth. After the common bile duct was cut off, the common pancreatic duct stent was removed and the split pancreatic was made (Fig. 1A). First, according to the size of the common pancreatic duct stent, a suitable size of the silicone catheter was selected as the internal stent and another relatively small size of the silicone catheter was selected as the external stent. The 2 silicone catheters were end-to-end and plug-in connected by 4-0 absorbable suture to form a split pancreatic duct stent (Figs. 1B and 2A). The internal stent was inserted into the pancreatic duct according to the depth of the previously placed common pancreatic duct stent, and the posterior wall of the PJ was sutured continuously using 3-0 or 4-0 Prolene suture (Fig. 1C). In the proximal jejunum, an electric hook was used to make a coarse incision with the internal stent (Fig. 1D), and the blind end of the jejunum was used to make a small incision with the external stent (Fig. 1E). Then, the external stent was drawn through the jejunum from the small incision (Fig. 1F), and the internal stent was partially introduced into the jejunum from the coarse incision (Fig. 1G). After the placement of the split pancreatic duct stent, 3-0 or 4-0 Prolene suture was used to perform PJ by purse-string suture, and the anterior wall of PJ was anastomosed by continuous suture. Finally, adjust the length of the external stent catheter and use 4-0 or 5-0 Prolene sutures to fix the external stent in the jejunum for tunnel embedding, and pull the external stent out of the abdominal wall through the cannula (Figs. 1H and 2B). After of 3 to 4 weeks, the suture at the junction was absorbed, the internal and external stent were separated, and the external stent was pulled out (Figs. 1I and 2C). An indwelling internal stent was used in vivo, to transform external drainage into internal drainage.

## 3. Results

The basic characteristics and postoperative pathology in all the patients are shown in Table 1. In this study, 12 patients (7 females and 5 males) were placed with split pancreatic drainage tube during LPD PJ. The average age of these patients was 63 years (range, 46–83 years), and the average BMI was 22.8 kg/m^2^ (range, 17.3–28.5 kg/m^2^). There were 2 ASA I, 7 ASA II and 3 ASA III patients. Postoperative pathological diagnoses included 8 pancreatic ductal adenocarcinoma, 3 cases of duodenal adenocarcinoma, and 1 case of distal cholangiocarcinoma patients.

**Table 1:** Patients’ basic characteristics and pathological diagnosis.

Variables	
Age, yr	63 ± 11.9
Gender (male/female)	5/7
BMI (kg/m^2^)	22.8 ± 3.3
American Society of Anesthesiology, n (%)	
I	2 (16.7)
II	7 (58.3)
III	3 (25.0)
Postoperative pathological diagnoses, n (%)	
Pancreatic ductal adenocarcinoma	8 (66.7)
Duodenal adenocarcinoma	3 (25.0)
Distal cholangiocarcinoma	1 (8.3)

The surgical results and complications in all the patients are shown in Table 2. The average operative time was 294.2 minutes (range, 230–350 minutes), average PJ time was 35.9 min (range, 30–45 minutes), and estimated blood loss was 204.2 mL (range, 120–300 mL). The average first exhaust time after surgery was 3.4 days (range, 2–5 days), The Postoperative hospitalization time was 10.7 days (range, 8–13 days), and no deaths occurred within 30 days after the surgery. Three of the 12 patients had postoperative complications, two of which were diagnosed as biochemical leaks and all improved after conservative treatment. One case had postoperative bleeding, which stopped after reoperation. During a median follow-up of 5 (3–8) months, no pancreatic leakage or other serious complications occurred.

**Table 2:** Results and postoperative complications.

Variables	
Operation time (min)	294.2 ± 36
Pancreaticojejunostomy time (min)	35.9 ± 4.1
Estimated blood loss (mL)	204.2 ± 58.2
Time of first postoperative venting (d)	3.4 ± 1
Postoperative hospitalization time (d)	10.7 ± 1.9
Death within 30 days, n (%)	0 (0)
Complications, n (%)	
POPF	2 (16.7)
Biochemical leakage	2 (16.7)
Grade B	0 (0)
Grade C	0 (0)
Biliary leakage	0 (0)
Postoperative bleeding	1 (8.3)
Delayed gastric emptying	0 (0)

POPF = postoperative pancreatic fistula.

## 4. Discussion

Owing to the continuous update of endoscopic equipment and development of an accurate minimally invasive concept, LPD has been widely used.^[20,21]^ However, owing to the deep anatomical position of the pancreas, the close connection with the surrounding organs and important blood vessels, and the long learning curve of LPD, the probability of complications, such as pancreatic leakage, biliary leakage, and postoperative bleeding after the LPD is still high.^[22]^ A recent multicenter randomized controlled trial showed that the incidence of severe complications after LPD was 29%, and 90-day mortality was 2%.^[23–25]^ POPF is the most common and serious complication after LPD, mainly due to leakage of pancreatic juice from pancreatic-jejunal anastomosis. Exuded pancreatic juice may lead to abdominal abscesses and bleeding, which seriously threatens the life of patients. To find the safest and most reliable method to reduce POPF, researchers have improved many anastomosis techniques, including^[26]^ direct end-to-side anastomosis,^[27]^ new catheter mucosal anastomosis (PJ),^[28]^ intrapped PJ,^[29]^ and combined PJ,^[30,31]^ but they do not seem to have achieved satisfactory results in reducing the incidence of POPF.

In recent years,^[11]^ studies have confirmed that drainage of pancreatic juice using pancreatic duct stents can effectively reduce the incidence of POPF and the risk of death after LPD.^[32]^ A prospective, multicenter randomized study conducted by Pessaux et al found that the incidence of POPF in patients with external stents (26%) was significantly lower than that in those without external stents (42%). The advantage of external stent is that the pancreatic juice is completely drained in vitro, which effectively reduces tension in the pancreatic jejunum anastomosis, and can also allow for the evaluation of the characteristics, color, and amount of drainage fluid. This is more convenient for determining the amylase content in the drainage fluid, so as to determine the patency of the drainage tube. However, there are many shortcomings of external drainage. In addition to the catheter-related complications such as catheter-related infection, accidental blockage, discounting and leakage, they also include adverse events, such as intestinal injury and leakage after removal of the external drainage catheter. Compared to external drainage, internal drainage appears more physiological.^[17]^ A retrospective study by Hirashita et al found that the incidence of POPF in patients with internal stents (16.5%) was lower than that those with external stents (42.3%). Internal drainage can effectively avoid the loss of a large number of electrolytes and trypsin caused by external drainage and nursing difficulties caused by external drainage, which can also cause complications, such as pancreatitis and intestinal obstruction due to stent movement, shedding, and bending.^[16,33–35]^ presently, there is still controversy over which type of internal and external stent drainage is the most effective.

To maximize the combination of internal and external drainage characteristics, we designed a new type of split drainage stent. Its main advantages include: the split pancreatic duct stent can convert external drainage into internal drainage, and the incidence of grade B or C POPF can be reduced to the greatest extent by combining the internal and external drainage characteristics; the split pancreatic duct stent can be designed according to the texture of the pancreas and size of the pancreatic tube to meet the need for different combinations of pancreatic drainage stent in patients, which can shorten the hospitalization time and improve the prognosis of patients; and the drainage stent is simple and widely applicable and does not require a long-term study by the surgeon. In our study, 2 patients had biochemical leakage, with an incidence of 16.7%, and no grade B or C. One case (8.3%) had postoperative bleeding, and no death occurred within 30 days after the operation. Therefore, we believe that this technique can effectively reduce the incidence of complications after LPD, especially grade B or C POPF.

## 5. Conclusion

The split pancreatic duct stent converts external stent drainage into internal stent drainage, which can effectively reduce the incidence of grade B or C POPF and other related complications. It provides additional pancreatic drainage methods for surgeons and has clinical value. However, the safety and efficacy of this drainage tube needs to be further validated in a larger prospective multicenter randomized controlled study.

## Author contributions

**Conceptualization:** Jianhua Tu.

**Data curation:** Jianhua Tu, Wenyan Xu.

**Investigation:** Shuaichang Gong, Zhenjun Cao, Ping Wan, Junxiang Ying.

**Methodology:** Changwen Huang, Xuefeng Rao.

**Software:** Jianhua Tu.

**Writing – original draft:** Jianhua Tu.

**Writing – review & editing:** Jianhua Tu, Zhenjun Cao, Ping Wan.

## Supplementary Material


